# Supplementary material for: Universal growth of perovskite thin monocrystals from high solute flux for sensitive self-driven X-ray detection
Source: Nat Commun. 2024 Mar 16;15:2390. doi: 10.1038/s41467-024-46712-y (PMC10944467; doi:10.1038/s41467-024-46712-y)
Supplement: Supplementary file 1 — Supplementary Information [file 41467_2024_46712_MOESM1_ESM.pdf]

Supplementary information

Universal growth of perovskite thin monocrystals from high solute flux for sensitive self-driven X-ray detection

Da Liu[1], Yichu Zheng[2], Xin Yuan Sui[1], Xue Feng Wu[1], Can Zou[1], Yu Peng[1], Xinyi Liu[1], Miaoyu Lin[1], Zhanpeng Wei[1], Hang Zhou[3], Ye-Feng Yao[3], Sheng Dai[4], Haiyang Yuan[1], Hua Gui Yang[1], Shuang Yang[1]* and Yu Hou[1]*

[1]Key Laboratory for Ultrafine Materials of Ministry of Education, Shanghai Engineering Research Center of Hierarchical Nanomaterials, School of Materials Science and Engineering, East China University of Science and Technology, 130 Meilong Road, 200237, Shanghai, China

[2]School of Mechatronic Engineering and Automation, Shanghai University, 99 Shangda Road, 200444, Shanghai, China

[3]Physics Department & Shanghai Key Laboratory of Magnetic Resonance, School of Physics and Electronic Science, East China Normal University, 3663 North Zhongshan Road, 200062, Shanghai, China

[4]Key Laboratory for Advanced Materials and Feringa Nobel Prize Scientist Joint Research Center, Institute of Fine Chemicals, School of Chemistry & Molecular Engineering, East China University of Science and Technology, 130 Meilong Road, 200237, Shanghai, China

*Correspondence: syang@ecust.edu.cn (S. Y.), yhou@ecust.edu.cn (Y. H.)

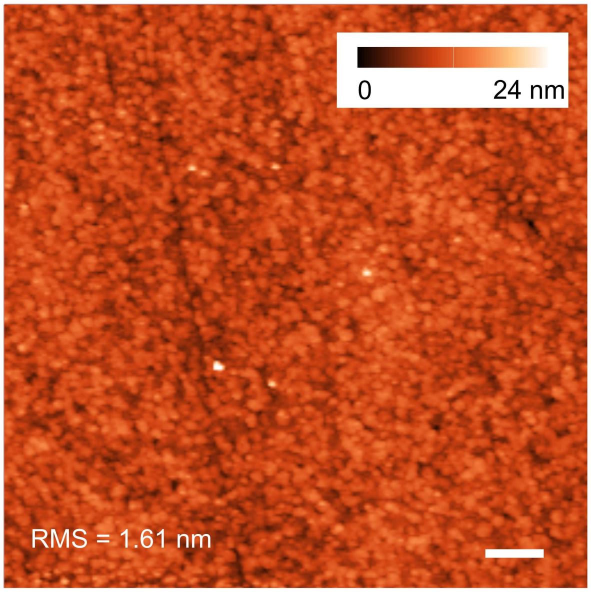

Supplementary Fig. 1 | Surface roughness of substrates. Atomic force microscope height image of the PTAA-covered ITO substrate. Scale bar: 1 μm.

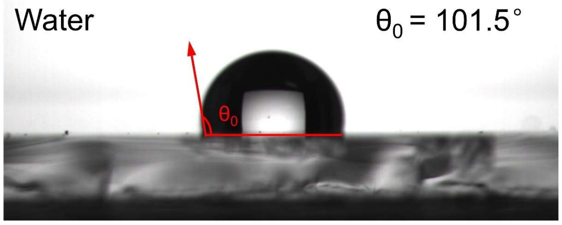

Supplementary Fig. 2 | Hydrophobicity of substrates. Contact angle measurements of the water on PTAA-covered ITO substrate.

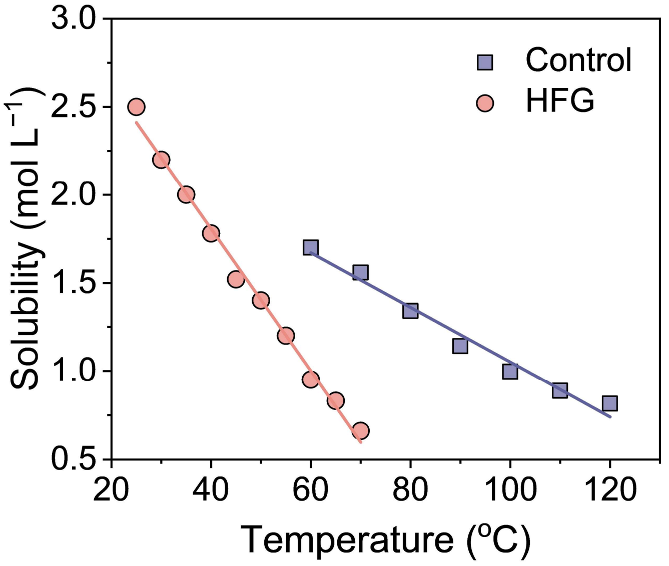

Supplementary Fig. 3 | Precursor solubility changes. Temperature-dependent solubility of $MAPbI_3$ precursors in different solvent. The precursor for high flux growth (HFG) shows a large solubility gradient with the increase of temperature.

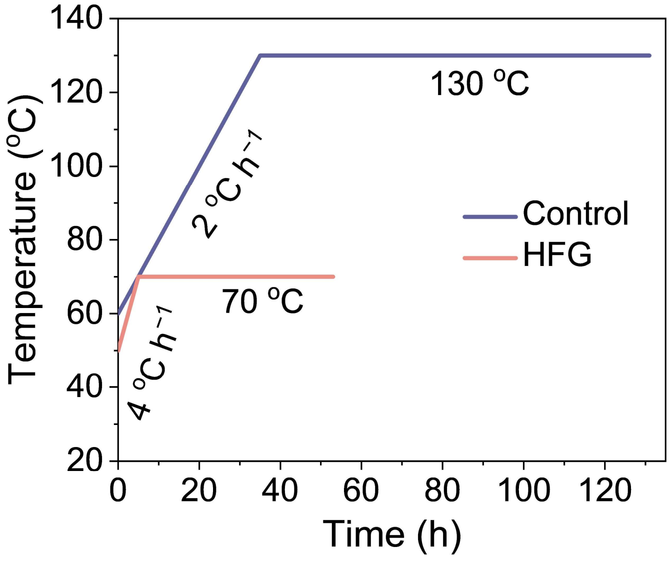

Supplementary Fig. 4 | Heating program. Heating curves of $MAPbI_3$ thin monocrystals from the control and high flux growth (HFG). The control sample is heated from 60 to 130 °C with a ramp rate of 2 °C h^{-1}, and the HFG sample (2 mol L^{-1}) is heated from 50°C to 70 °C with a heating rate of 4 °C h^{-1}.

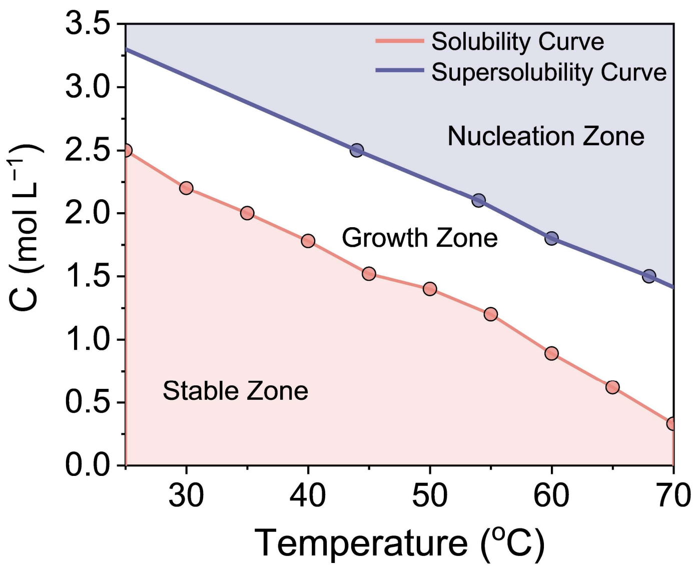

Supplementary Fig. 5 | The dissolution-nucleation diagram. The whole region is separated by the solubility and the supersaturation curves.

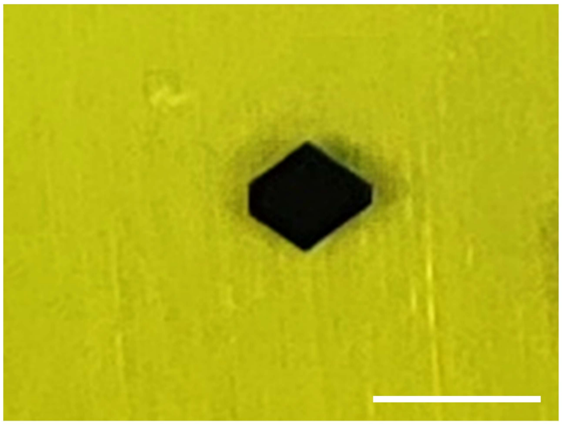

Supplementary Fig. 6 | The photograph of the control thin monocrystal. Scale bar: 5 mm.

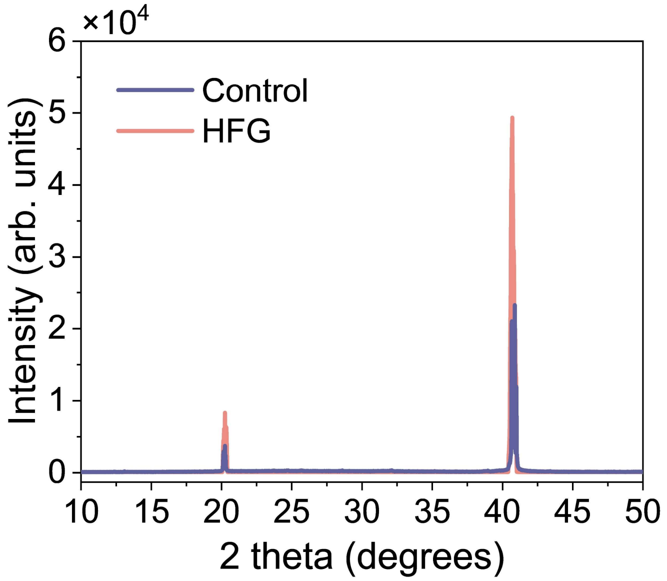

Supplementary Fig. 7 | Crystallinity of monocrystals. XRD patterns of thin monocrystals from the control and high flux growth (HFG).

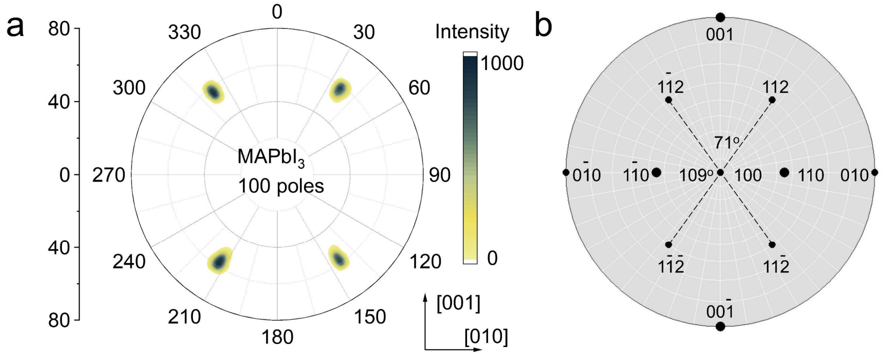

Supplementary Fig. 8 | Pole figure measurement. a, Pole figure along the (400) orientation of the HFG thin monocrystal. **b**, Simulated pole figure of space group of *I*4 *cm* from the 100 Bragg reflection, which has a cell length of a = 8.859 Å, b = 8.859 Å, c = 12.649 Å.

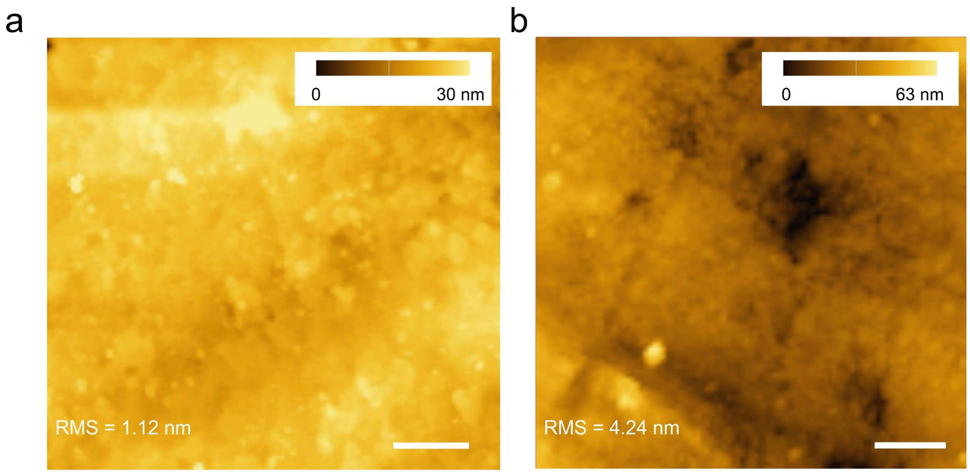

Supplementary Fig. 9 | Surface roughness of monocrystals. Atomic force microscope height image of thin monocrystals from **a** high flux growth (HFG) and **b** control. Scale bars: 1 μm.

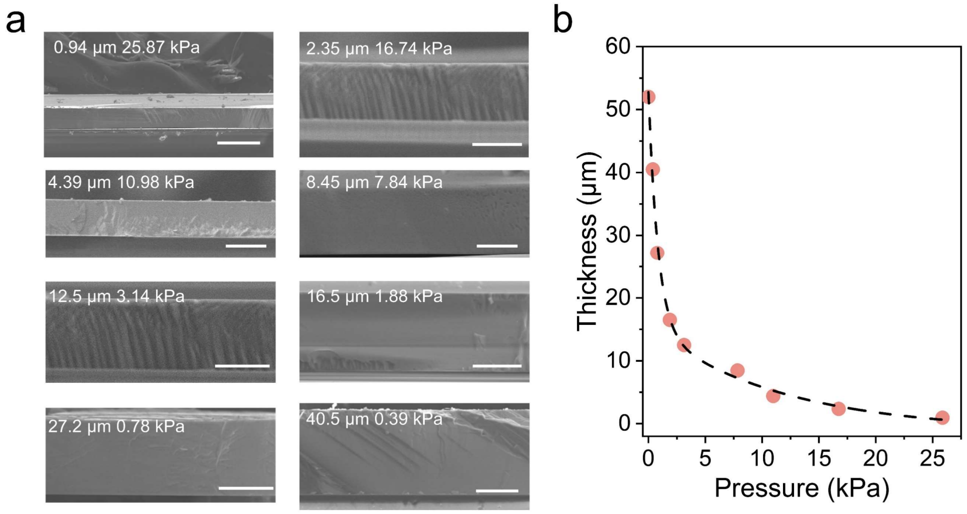

Supplementary Fig. 10 | Tunable thickness of monocrystal. a, Cross-sectional SEM images of the HFG $MAPbI_3$ thin monocrystals under different pressure. Scale bar: 2 μm for 25.87 and 16.74 kPa, 5 μm for 10.98 and 7.84 kPa, 10 μm for 3.14 and 1.88 kPa, 20 μm for 0.78 and 0.39 kPa. **b**, Thickness of thin monocrystals as a function of pressure.

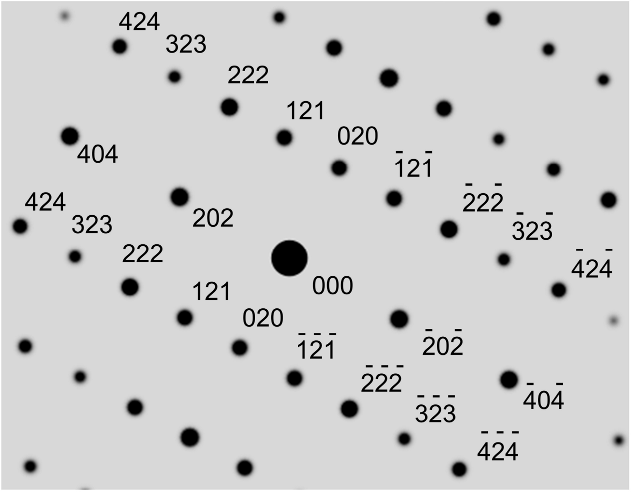

Supplementary Fig. 11 | Simulated electron diffraction patterns. The tetragonal $MAPbI_3$ with the space group of *I*4 *cm* along [$\bar{1}$01] axis zone has a cell length of a = 8.859 Å, b = 8.859 Å, c = 12.649 Å.

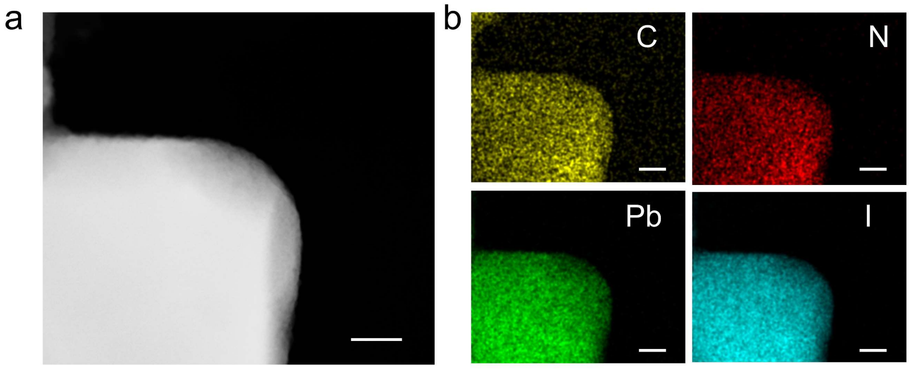

Supplementary Fig. 12 | Element distribution. a, High angle annular dark field (HAADF)-STEM images of the tetragonal $MAPbI_3$ thin monocrystal from high flux growth. **b**, EDX analysis shows the elemental distribution of C, N, Pb, and I. Scale bars: 100 nm.

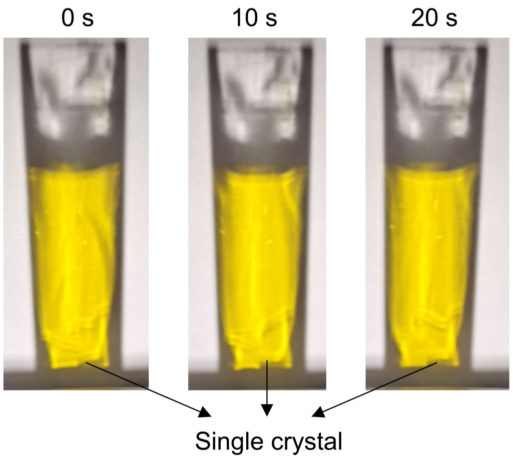

Supplementary Fig. 13 | Inhomogeneous concentration distribution. Projection map of perovskite precursor solution at different times during the solution process of perovskite monocrystal.

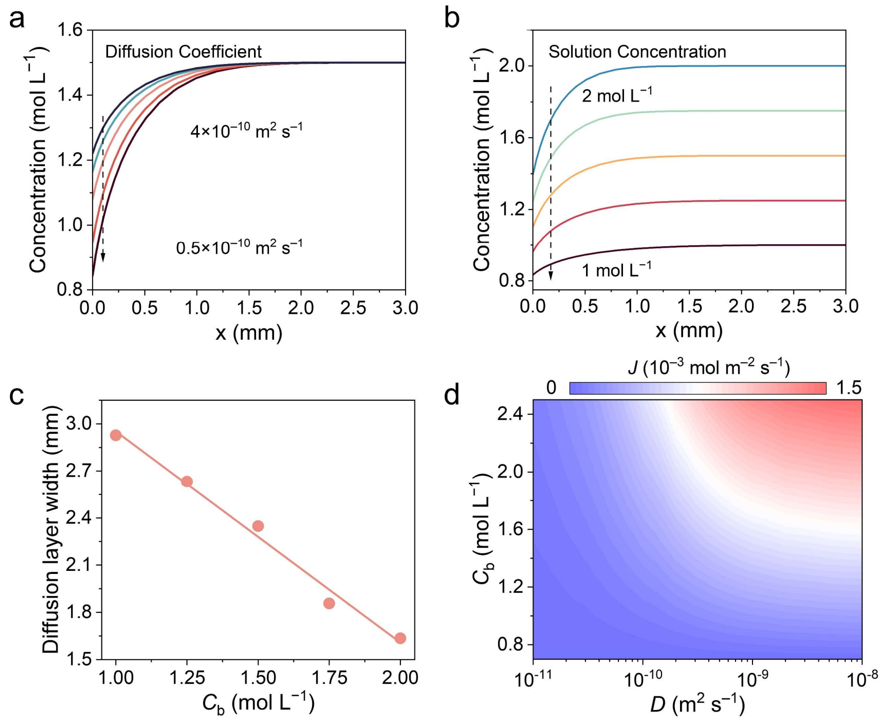

Supplementary Fig. 14 | Simulated concentration field. a, Simulated concentration field of solute near a $MAPbI_3$ monocrystal with varied diffusion coefficients (0.5–4×10^{10} m^2 s^{-1}). **b**, Simulated concentration field of solute near a $MAPbI_3$ monocrystal with varied initial concentration (1–2 mol L^{-1}). **c**, Variation of diffusion layer width with initial concentration. The increase in the bulk concentration (C_b) leads to a decrease in the diffusion layer width, which further promotes the diffusion of the monomer. **d**, Effect of C_b and diffusion coefficients (D) on the growth flux (J) of perovskite thin monocrystal.

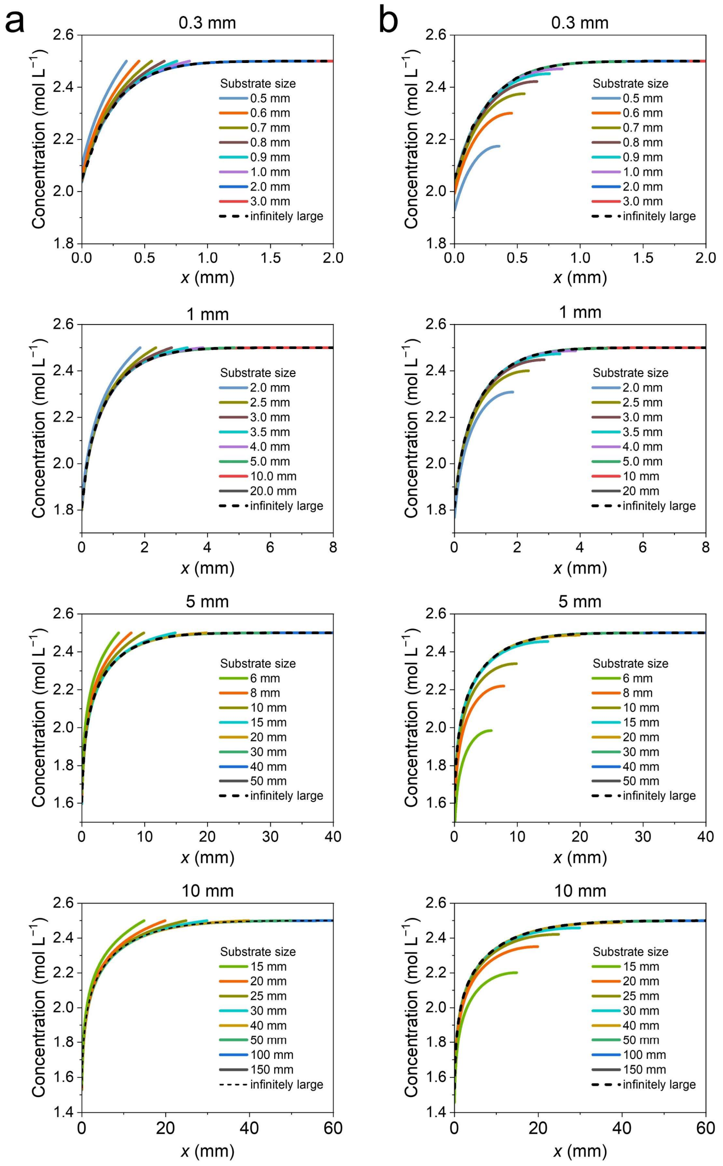

Supplementary Fig. 15 | Simulated concentration distribution. Simulated concentration field of solute near a $MAPbI_3$ monocrystal **a** with and **b** without the boundary condition of $x = r_s$, $C = C_b$, where r_s is the radius of substrate, C_b is bulk solution concentration. The monocrystal size grows from 150 μm to a certain size, i.e., 300 μm, 1 mm, 5 mm, and 1 cm. The diffusion coefficient is 5×10^{-10} m^2 s^{-1} and the initial solution concentration is 2.5 mol L^{-1}. For both conditions with different final crystal radius (r_c), the concentration distribution approaches the one simulated under infinite large substrate as r_s increases, and becomes almost undistinguishable when $r_s/r_c > 3$.

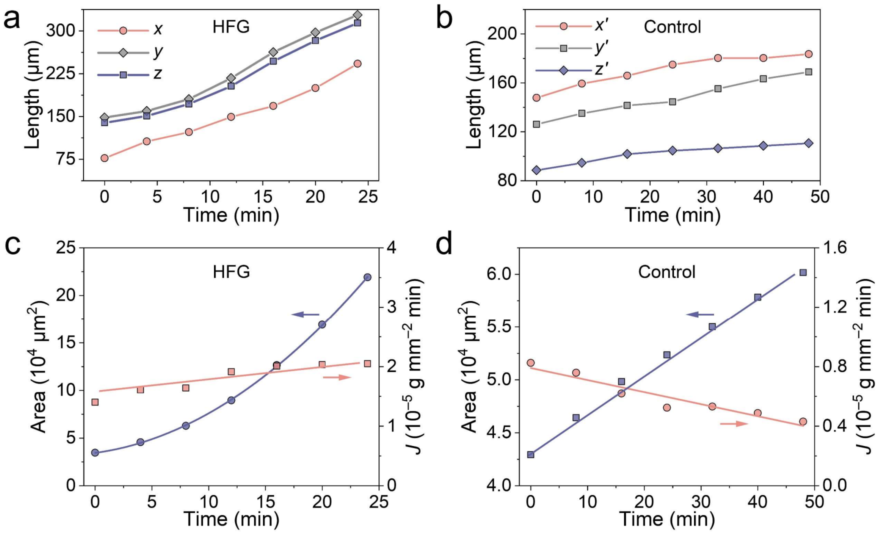

Supplementary Fig. 16 | The size changes of monocrystals during growth process. Evolution of the length of $MAPbI_3$ thin monocrystals from **a** high flux growth (HFG) and **b** control. The area and solute flux (*J*) as a function of the growth time for the **c** HFG and **d** control $MAPbI_3$ thin monocrystals. During the entire monitoring period, the length of HFG monocrystal exhibits a nearly linear growth trend. The area of HFG monocrystal is fitted by a quadratic function, indicating a stable solute flux. In contrast, the area of control monocrystal displays a nearly linear growth trend due to insufficient solute supply.

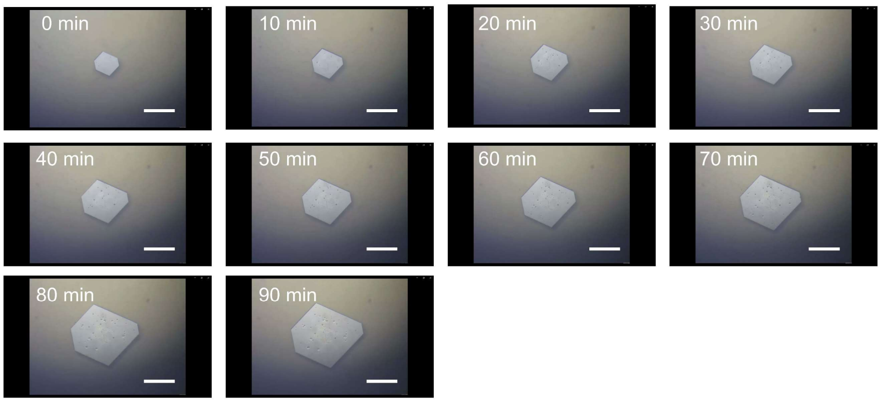

Supplementary Fig. 17 | Optical images of monocrystal during growth process. Optical images of the growth process of $MAPbI_3$ thin monocrystal from high flux growth. Scale bar is 1 mm.

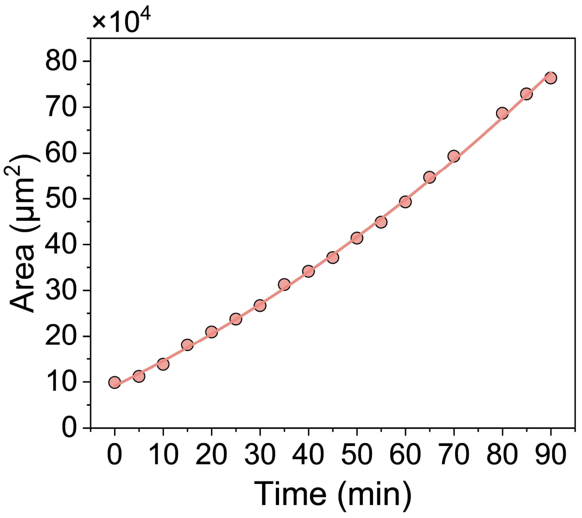

Supplementary Fig. 18 | The size changes of monocrystal during growth process. Monocrystal area as a function of the growth time for the $MAPbI_3$ thin monocrystal from high flux growth (HFG). The area of HFG monocrystal can be fitted by a quadratic function, indicating a stable solute flux.

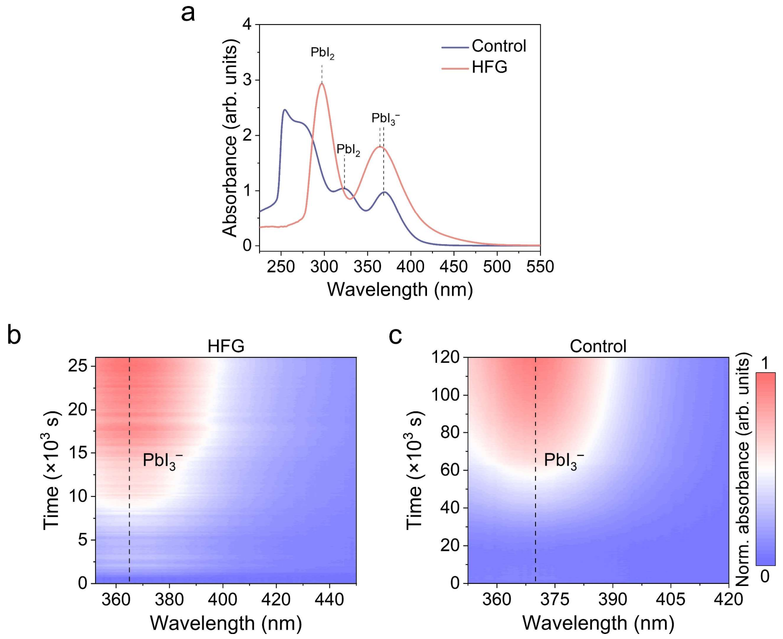

Supplementary Fig. 19 | Absorption spectra change during the diffusion process. **a**, UV−vis absorption spectra of the $MAPbI_3$ precursor solutions. Evolution of UV−vis absorption of solvents for **b** high flux growth (HFG) and **c** control after injection 20 μL of the diluted perovskite precursor solutions at the bottom of the cuvette.

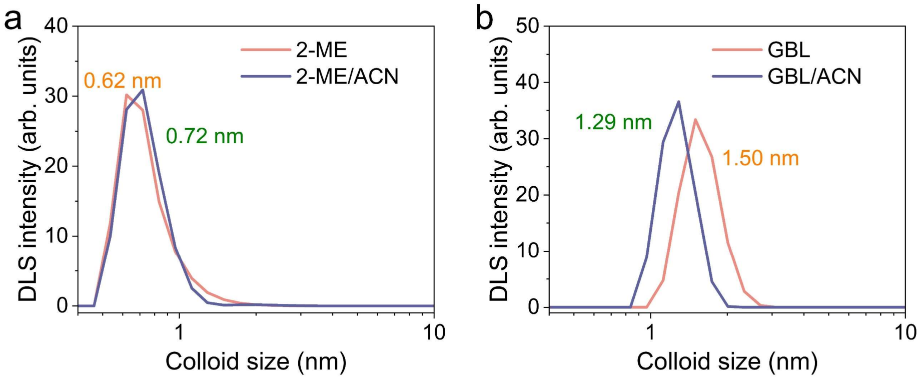

Supplementary Fig. 20 | Effect of acetonitrile addition on perovskite colloids. Colloidal size of **a** 2-ME and **b** GBL solution via dynamic light scattering after introducing deuterated ACN at a volume ratio of 10%.

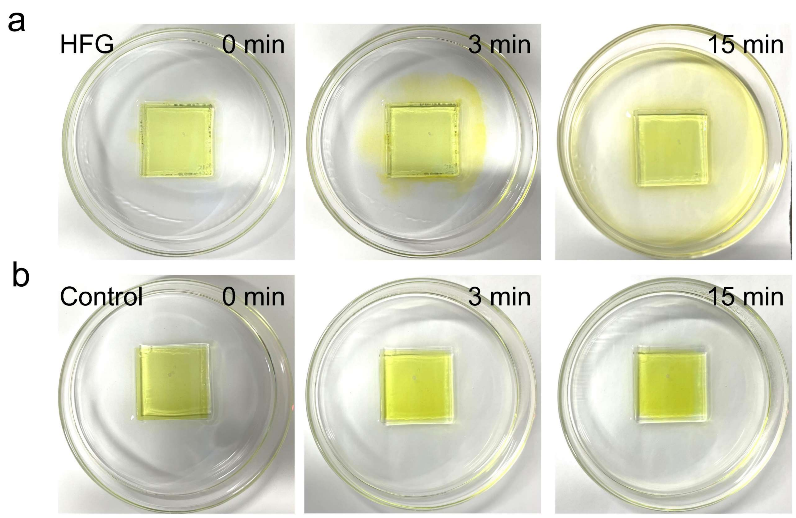

Supplementary Fig. 21 | Snapshots of the diffusion process. Diffusion process of perovskite precursors to pure solvents for **a** high flux growth (HFG) and **b** control. The precursor solution is confined by two glass substrates. The temperature of the HFG and control samples are 70 and 130°C, respectively.

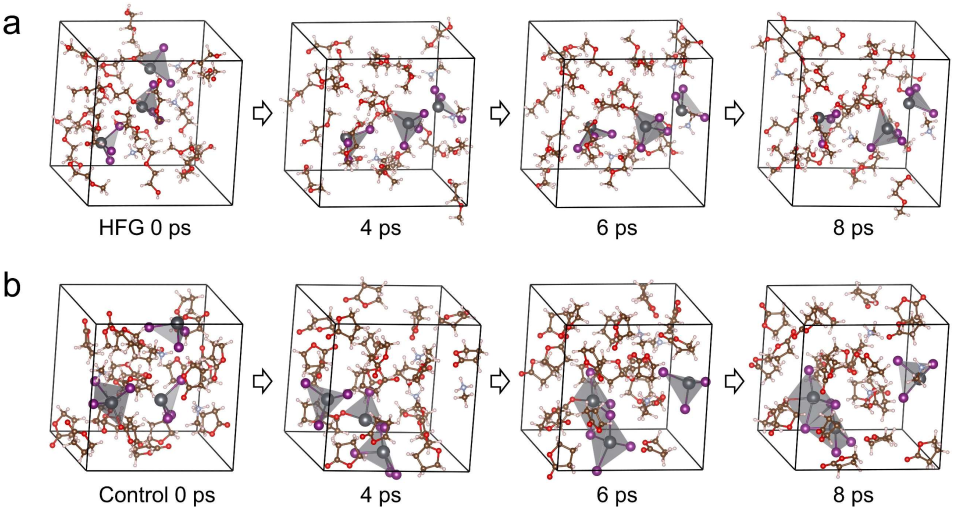

Supplementary Fig. 22 | Ab initio MD simulations. Ab initio MD simulations of **a** high flux growth (HFG) and **b** control model systems. The colors of the atoms are cyan: Pb, purple: I, brown: C, pink: H, light purple: N, red: O. All species are shown with ball and stick representations.

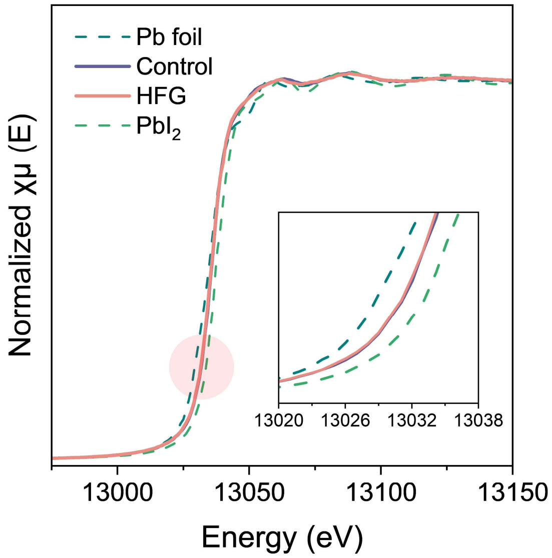

Supplementary Fig. 23 | X-ray absorption near edge structure. Pb L_3-edge X-ray absorption near edge structure spectra of perovskite solution for the control and high flux growth (HFG), reference Pb foil, and PbI_2 sample. The inset shows the magnified cutoff region.

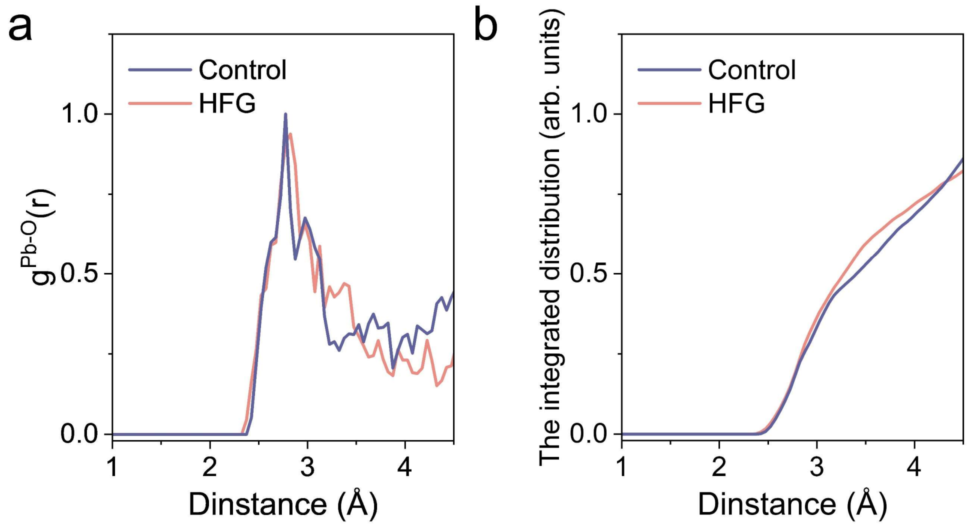

Supplementary Fig. 24 | Extended X-ray absorption fine structure. a, Pb−O radial distribution functions (g^{Pb-O}) and **b** the integrated distribution of high flux growth (HFG) and control model systems, averaged over the 10 ps.

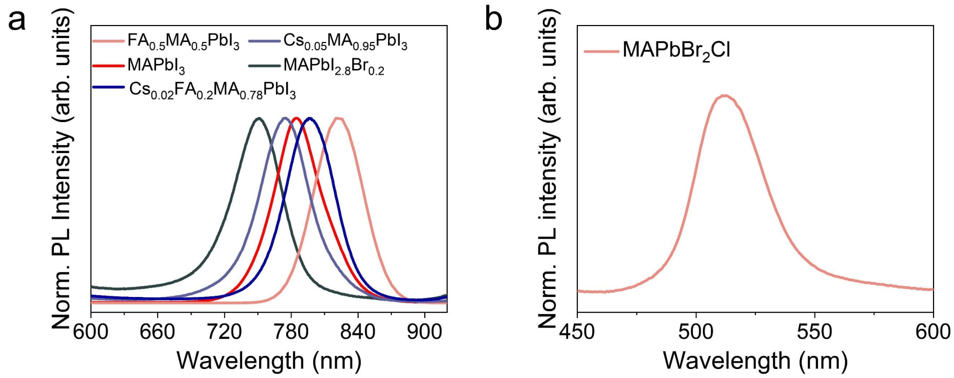

Supplementary Fig. 25 | Photoluminescence measurement of perovskite alloys. a Steady-state photoluminescence (PL) spectra of thin monocrystals of perovskite alloys. **b** PL spectrum of $MAPbBr_2Cl$ thin monocrystal. The excitation wavelength is 365 nm.

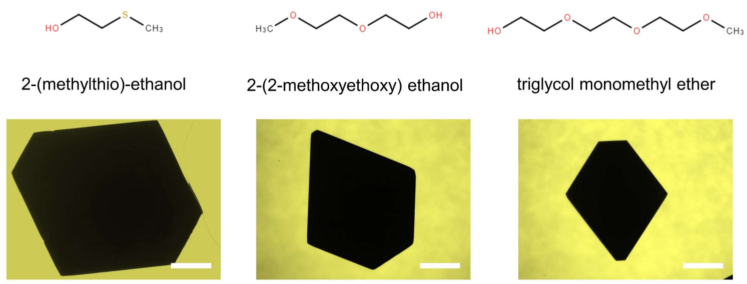

Supplementary Fig. 26 | Different glycol ether solvents for growing monocrystal. Molecular structure of glycol ether solvents similar to that of 2-methoxyethanol. Optical microscope images of $MAPbI_3$ thin monocrystal grown from glycol ether solvents for 24 h. Scale bars: 300 μm.

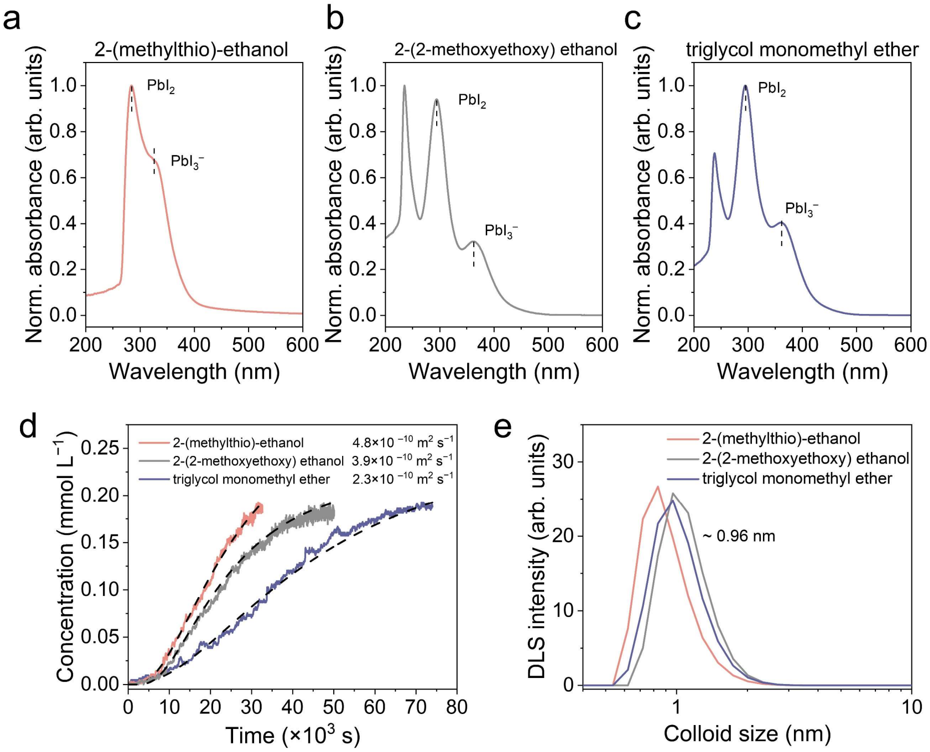

Supplementary Fig. 27 | Diffusion behavior of different glycol ether solvents. a−c, UV−vis absorption spectra of the $MAPbI_3$ dissolved in different glycol ether solvents. **d**, Variation in time-dependent concentration of perovskite precursors that diffuse from the bottom of cuvette. **e**, Colloidal hydrodynamic size distribution via dynamic light scattering of perovskite precursor solutions. Perovskite precursor in these glycol ether solvents exhibits small micelle diameters as well.

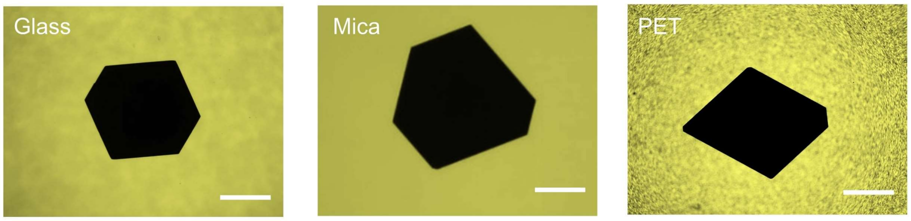

Supplementary Fig. 28 | Monocrystals grown on different substrates. Optical microscope images of the $MAPbI_3$ thin monocrystal grown on different substrates. Scale bars: 500 µm.

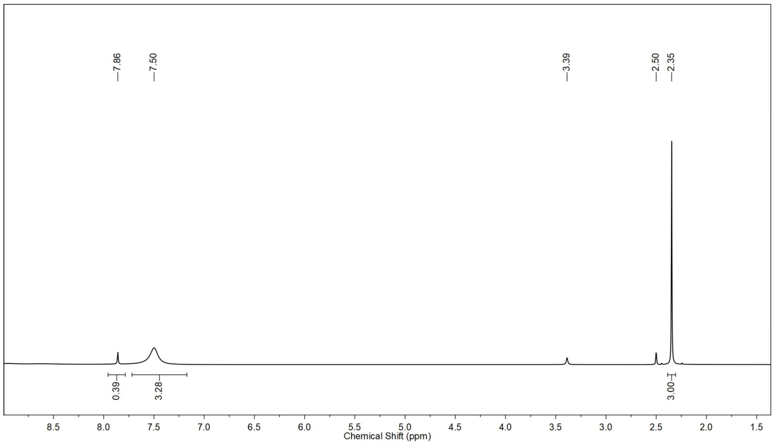

Supplementary Fig. 29 | NMR measurement. ^{1}H NMR spectroscopy of the triple-cation perovskite monocrystal (DMSO- D_6, 500 MHz).

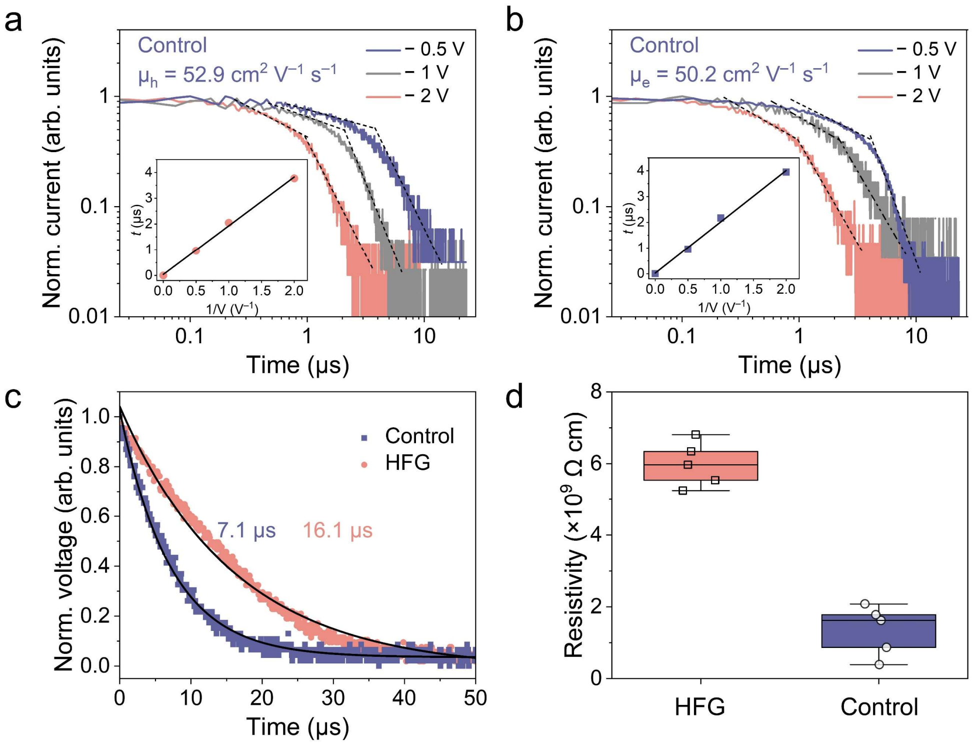

Supplementary Fig. 30 | Optoelectronic properties of $Cs_{0.02}FA_{0.2}MA_{0.78}PbI_3$ thin monocrystals. Normalized time-of-flight **a** hole and **b** electron charge transient current curves of the control thin monocrystal device under various reverse bias voltages. The inset shows the charge transit time versus the reciprocal of bias voltage. The excitation wavelength is 337 nm. **c**, Transient photovoltage decay curves of thin monocrystal devices. The carrier lifetime of the device from high flux growth (HFG) is determined to be 16.1 μs, which is longer than that of the control device (7.1 μs). **d**. The resistivity of $Cs_{0.02}FA_{0.2}MA_{0.78}PbI_3$ thin monocrystals.

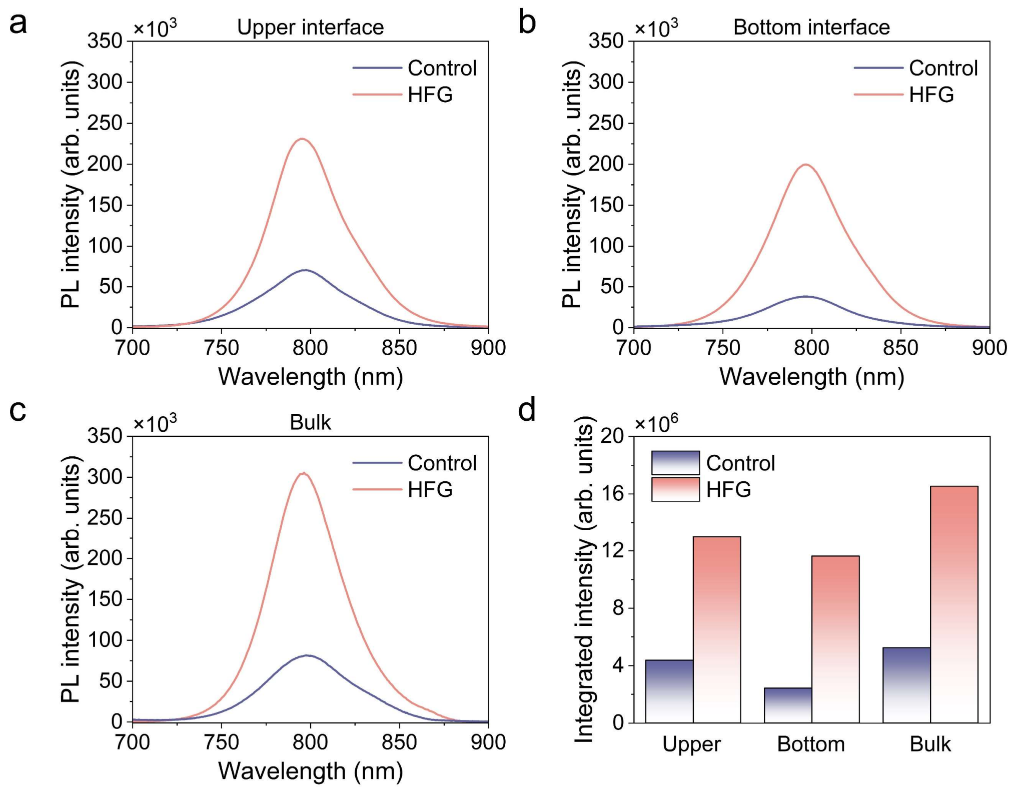

Supplementary Fig. 31 | Photoluminescence measurement of different depth. Steady-state photoluminescence (PL) spectra of $Cs_{0.02}FA_{0.2}MA_{0.78}PbI_3$ thin monocrystals from the control and high flux growth (HFG). A 365 nm LED laser is employed to evaluate the defect-induced recombination of the **a** upper and **b** bottom interfaces of thin monocrystals. The excitation wavelength of **c** the bulk PL spectra of thin monocrystals is 635 nm. **d**, Comparison of the integrated PL intensity of thin monocrystals.

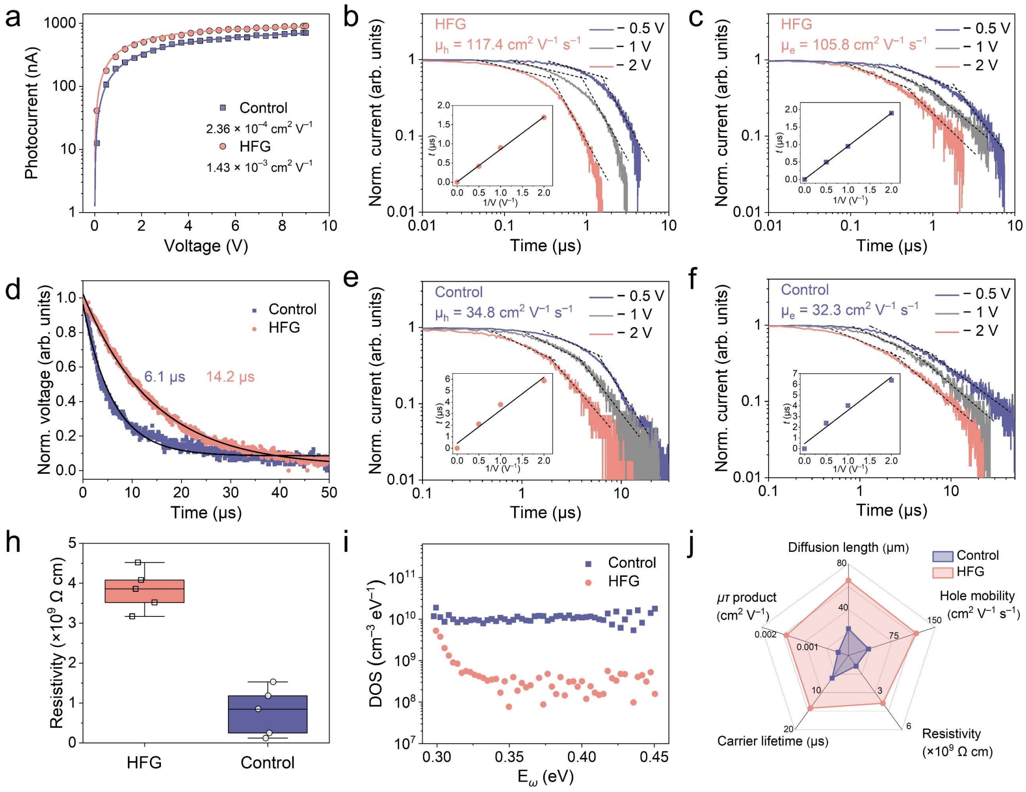

Supplementary Fig. 32 | Optoelectronic properties of $MAPbI_3$ thin monocrystals. **a**, Photoconductivity measurement of thin monocrystal devices. Normalized time-of-flight **b** hole and **c** electron charge transient current curves of thin monocrystal device from the control and high flux growth (HFG) under various reverse bias voltages. The inset shows the charge transit time versus the reciprocal of bias voltage. **d**, Transient photovoltage decay curves of thin monocrystal devices. The carrier lifetime of the HFG device is determined to be 14.2 µs, which is longer than that of the control device (6.1 µs). Normalized time-of-flight **e** hole and **f** electron charge transient current curves of the control thin monocrystal device under various reverse bias voltages. The inset shows the charge transit time versus the reciprocal of bias voltage. **h**. The resistivity of $MAPbI_3$ thin monocrystals. **i**, Trap density of states of thin monocrystal devices. **j**, Radar chart comparing diffusion length, hole mobility, resistivity, carrier lifetime, and $\mu\tau$ product for testing the optoelectronic properties of $MAPbI_3$ thin monocrystals.

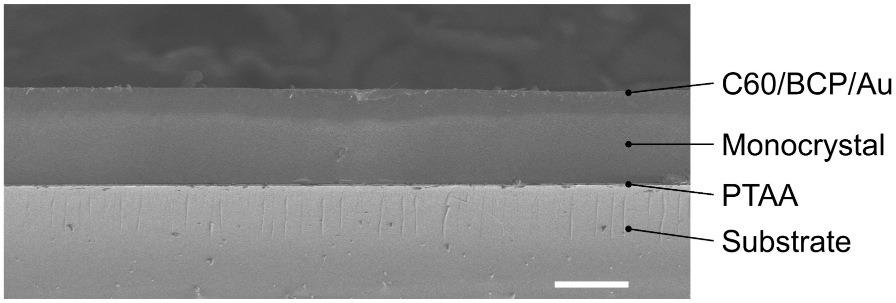

Supplementary Fig. 33 | Device structure. Cross-sectional SEM image of the self-driven thin monocrystal device. Scale bar: 50 µm.

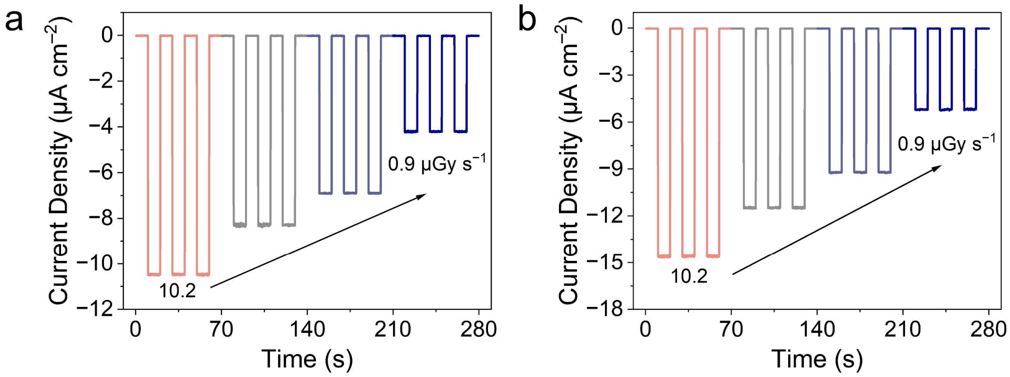

Supplementary Fig. 34 | X-ray response of HFG device at bias voltage. Time-dependent response of $Cs_{0.02}FA_{0.2}MA_{0.78}PbI_3$ thin monocrystal device from high flux growth (HFG) under **a** 1 V and **b** 1.5 V bias.

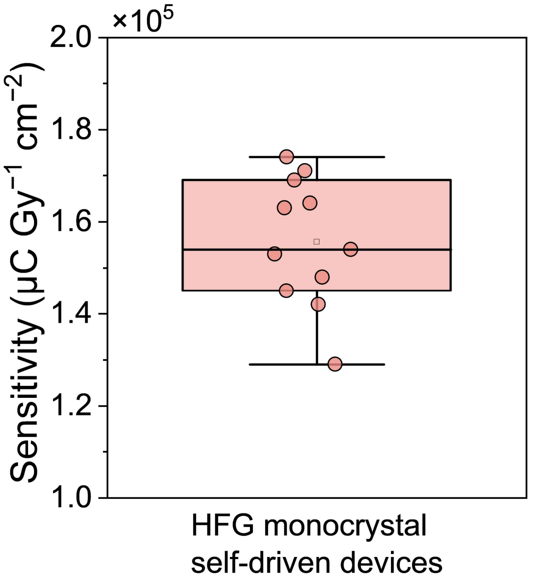

Supplementary Fig. 35 | Sensitivity distribution. Sensitivity distribution of self-driven devices from high flux growth (HFG).

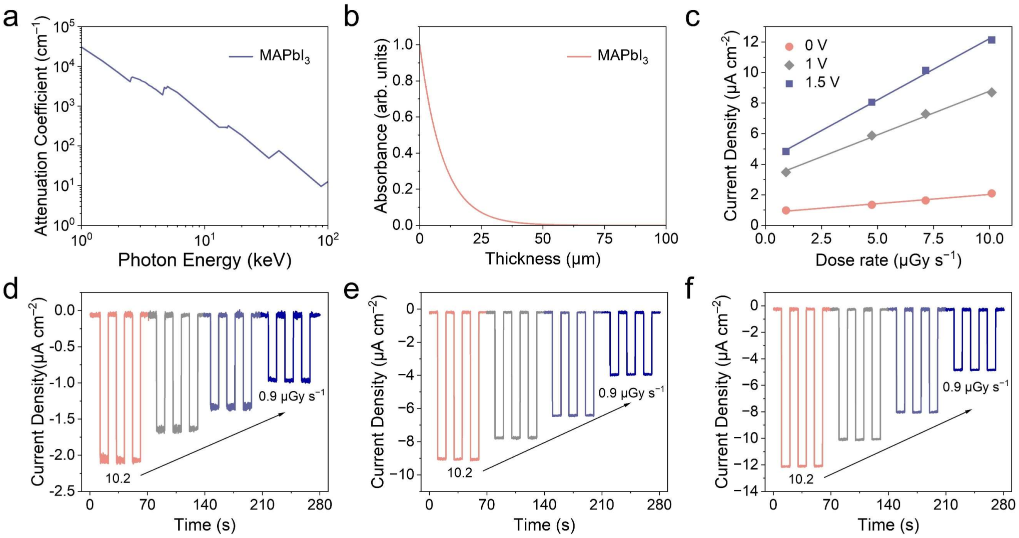

Supplementary Fig. 36 | X-ray detection performance of the $MAPbI_3$ thin monocrystal device. **a**, The calculated attenuation coefficient of $MAPbI_3$ to 8 keV X-ray photons. **b**, The calculated attenuation efficiency of $MAPbI_3$ to 8 keV X-ray photons. **c**, The output current density of thin monocrystal device from high flux growth (HFG) under different dose rates. Time-dependent response of thin monocrystal device under **d** 0 V, **e** 1 V, and **f** 1.5 V bias.

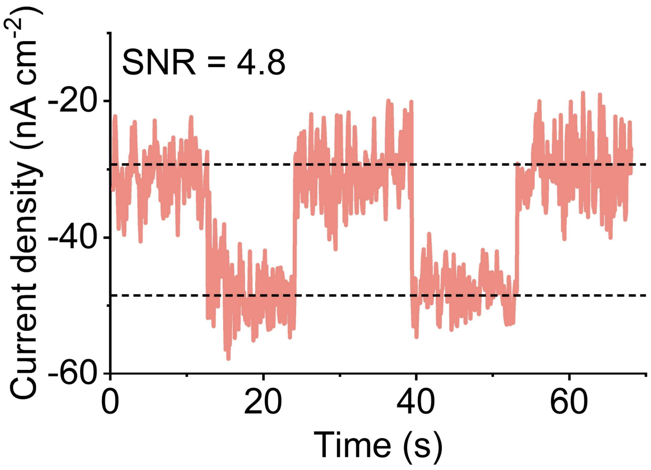

Supplementary Fig. 37 | X-ray response at low doses. X-ray response of thin monocrystal device from the high flux growth at a low dose of 14.6 nGy s^{-1}.

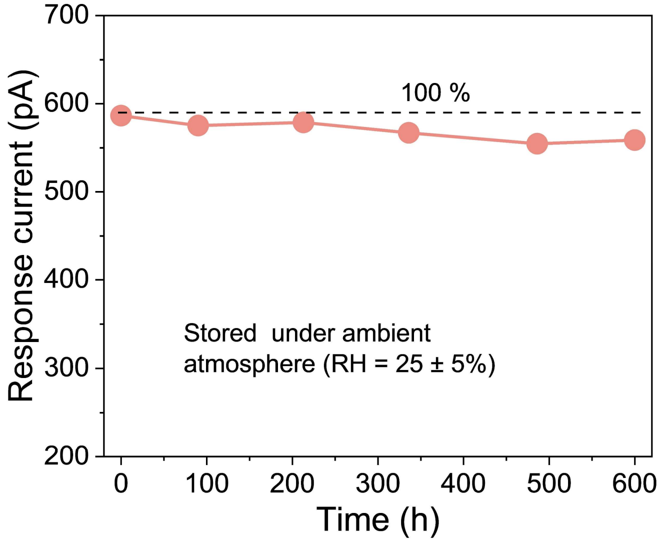

Supplementary Fig. 38 | Long term stability. Response current evolution of the unencapsulated self-driven device from high flux growth under ambient atmosphere (RH = 20 ± 5%).

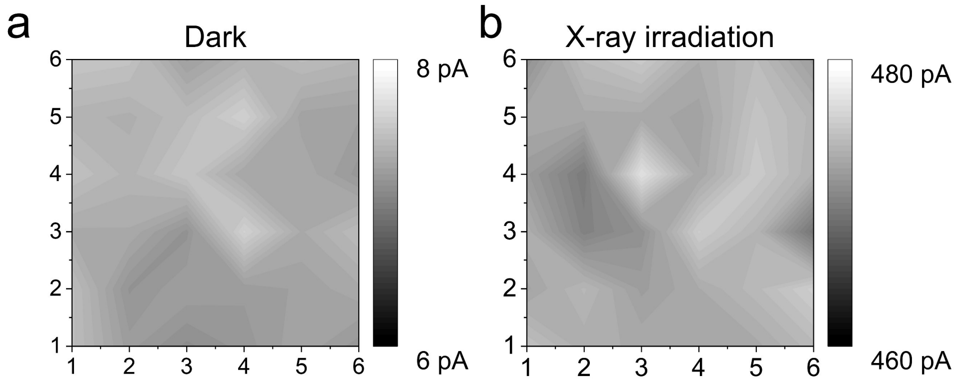

Supplementary Fig. 39 | Uniformity of the arrayed devices. Current of self-driven devices (6×6 pixel array) in **a** dark and under **b** X-ray irradiation.

Supplementary Table 1 | Average bond length of Pb-I in the 2-ME and GBL solvent molecules

Time	$Pb\text{-}I_{2\text{-}ME}$(Å)	$Pb\text{-}I_{GBL}$(Å)
**5 ps**	3.027	3.126
**7.5 ps**	3.084	3.088
**10 ps**	3.097	3.155

Supplementary Table 2 | Summary of the synthesis temperature of monocrystal.

Perovskite	Bandgap	This work	Other report	References
**$MAPbI_3$**	1.59	70 ℃	130 ℃	[1]
**$BA_2MAPb_2I_7$**	2.2	90 ℃	130 ℃	[2]
**$FASnI_3$**	1.37	60 ℃	80 ℃	[3]
**$MAGeI_3$**	1.90	40 ℃	120 ℃	[4]
**$FAGeI_3$**	2.20	40 ℃	120 ℃	[4]
**PEA_2PbI_4**	2.28	70 ℃	80 ℃	[5]
**$BDAPbI_4$**	2.37	70 ℃	90 ℃	[6]
**$Cs_3Sb_2I_9$**	2.34	60 ℃	120 ℃	[7]
**MA_2AgSbI_6**	1.93	70 ℃	150 ℃	[8]
**$Cs_3Bi_2I_9$**	2.50	60 ℃	70 ℃	[9]
**$MA_3Bi_2I_9$**	2.00	60 ℃	100 ℃	[10]
**$FA_3Bi_2I_9$**	2.08	60 ℃	70 ℃	[11]
**$Cs_3Cu_2I_5$**	2.79	50 ℃	60 ℃	[12]
**$Cs_2AgBiBr_6$**	1.79	60 ℃	120 ℃	[13]
**PEA_2PbBr_4**	3.03	40 ℃	70 ℃	[14]
**$FAPbBr_3$**	2.26	50 ℃	65 ℃	[15]
**$MAPbBr_3$**	2.20	50 ℃	120 ℃	[16]
**$MAPbCl_3$**	3.00	50 ℃	100 ℃	[17]

Supplementary Table 3 | Summary of the sensitivity and lowest detectable dose rate of self-driven X-ray detectors.

Materials	Bias voltage (V)	Sensitivity (μC Gy cm^{-2})	Lowest detectable dose rate （nGy s^{-1}）	References
**GaN**	0	0.17	N/A	[18]
**$(BA)_2CsAgBiBr_7$ /$Cs_2AgBiBr_6$ monocrystal**	0	206	N/A	[19]
**PbI_2 monocrystal**	0	1200	N/A	[20]
**$FA_{0.55}MA_{0.45}PbI_3$ thin monocrystal**	0	80000	27.7	[21]
**$MAPbI_3$ thin monocrystal**	0	126160	N/A	this work
**$Cs_{0.02}FA_{0.2}MA_{0.78}PbI_3$ thin monocrystal**	0	173580	11.8	this work

Supplementary Table 4 | Summary of the sensitivity and lowest detectable dose rate of X-ray detectors.

Materials	Bias voltage (V)	Sensitivity (μC Gy cm^{-2})	Lowest detectable dose rate （nGy s^{-1}）	References
**(CsFAGA:Sr)Pb ($I_{0.1}Br_{0.9}$)$_3$ monocrystal**	0.17	27083	7.09	[22]
**$MAPbI_3$ monocrystal**	1	968.9	N/A	[23]
**$(NH_4)_3Bi_2I_9$ monocrystal**	1	8200	55	[24]
**$CsPbBr_3$ monocrystal**	5	2549.36	1890	[25]
**$GAMAPbI_3$ monocrystal**	5	23000	16.9	[26]
**$MAPbBr_3$/Si**	7	21000	36	[27]
**$BA_2EA_2Pb_3Br_{10}$ monocrystal**	10	6800	N/A	[28]
**$MAPbI_3$ thin monocrystal**	1.5	784890	N/A	this work
**$Cs_{0.02}FA_{0.2}MA_{0.78}PbI_3$ thin monocrystal**	1.5	1013360	N/A	this work

Supplementary Table 5 | Summary of the growth parameters of thin monocrystals based on 2-ME solvent.

Type	Perovskite	Space group	Ramp rate	Temp./Time	Growth rate (μm² h⁻¹)
3D	$MAPbI_{2.8}Br_{0.2}$	Tetragonal (*I*4*mcm*)	5 °C h^{-1}	70 °C/48 h	3.60×10^5
	$FA_{0.5}MA_{0.5}PbI_3$	/	8 °C h^{-1}	90 °C/48 h	2.83×10^5
	$Cs_{0.05}MA_{0.95}PbI_3$	Tetragonal (*I*4*mcm*)	5 °C h^{-1}	90 °C/48 h	3.31×10^5
	$Cs_{0.02}FA_{0.2}MA_{0.78}PbI_3$	Tetragonal (*I*4*mcm*)	5 °C h^{-1}	90 °C/48 h	3.15×10^5
	$MASnI_3$	Cubic (*Pm*-3*m*)	3 °C h^{-1}	60 °C/24 h	2.73×10^5
	$FASnI_3$	Cubic (*Pm*-3*m*)	3 °C h^{-1}	60 °C/24 h	2.00×10^5
	$MAGeI_3$	Trigonal (*R*3*m*)	3 °C h^{-1}	40 °C/36 h	7.54×10^4
	$FAGeI_3$	Trigonal (*R*3*m*)	3 °C h^{-1}	40 °C/24 h	3.92×10^4
	$CsGeI_3$	Trigonal (*R*3*m*)	3 °C h^{-1}	40 °C/24 h	2.71×10^4
	MA_2AgSbI_6	Orthorhombic	3 °C h^{-1}	70 °C/48 h	9.96×10^4
	MA_2AgBiI_6	Orthorhombic	3 °C h^{-1}	70 °C/48 h	1.19×10^5
	$Cs_2AgBiBr_6$	Cubic (*Fm*-3*m*)	3 °C h^{-1}	60 °C/24 h	3.79×10^4
2D (quasi 2D)	BA_2PbI_4	Orthorhombic (*Pbca)*	8 °C h^{-1}	80 °C/72 h	9.12×10^4
	PEA_2PbI_4	Triclinic (*P*-1*)*	8 °C h^{-1}	70 °C/72 h	6.17×10^4
	$BDAPbI_4$	Triclinic (*P*1)	8 °C h^{-1}	70 °C/72 h	5.89×10^4
	PEA_2PbBr_4	Triclinic (*P*-1*)*	3 °C h^{-1}	40 °C/72 h	5.69×10^4
	$BA_2MAPb_2I_7$	Orthorhombic (*Cc*2*m*)	3 °C h^{-1}	90 °C/96 h	1.44×10^5

Supplementary Table 5 (continued) | Summary of the growth parameters of thin monocrystals based on 2-ME solvent.

Type	Perovskite	Space group	Ramp rate	Temp./Time	Growth rate ($\mu m^2\ h^{-1}$)
**1D**	**$DMAPbI_3$**	Hexagonal ($P6_3/mmc$)	10 °C h^{-1}	70 °C/24 h	1.10×10^5
**0D**	**$Cs_3Sb_2I_9$**	Hexagonal ($P6_3/mmc$)	3 °C h^{-1}	60 °C/24 h	4.34×10^4
	**$MA_3Sb_2I_9$**	Hexagonal ($P6_3/mmc$)	3 °C h^{-1}	60 °C/36 h	8.11×10^4
	**$Cs_3Bi_2I_9$**	Hexagonal ($P6_3/mmc$)	3 °C h^{-1}	60 °C/36 h	3.90×10^4
	**$MA_3Bi_2I_9$**	Hexagonal ($P6_3/mmc$)	3 °C h^{-1}	60 °C/36 h	1.03×10^5
	**$FA_3Bi_2I_9$**	Hexagonal ($P6_3mc$)	3 °C h^{-1}	60 °C/36 h	8.33×10^4
	**$Cs_3Cu_2I_5$**	Orthorhombic (*Pnma*)	10 °C h^{-1}	50 °C/36 h	1.56×10^4

Supplementary Table 6 | Summary of the growth parameters of thin monocrystals based on the mixed solvent.

Perovskite	Solvent	Space group	Ramp rate	Temp./Time	Growth rate ($\mu m^2\ h^{-1}$)
**$FAPbBr_3$**	DMF/GBL/2-ME 1:1:1 (volume ratio)	Cubic (*Pm*-3*m*)	4 °C/h	50 °C/48 h	5.26×10^4
**$MAPbBr_3$**	DMF/2-ME 1:1 (volume ratio)	Cubic (*Pm*-3*m*)	4 °C/h	50 °C/36 h	7.01×10^4
**$MAPbBr_2Cl$**	DMF/2-ME 4:1 (volume ratio)	Cubic (*Pm*-3*m*)	4 °C/h	40 °C/48 h	3.51×10^4
**$MAPbCl_3$**	DMSO/2-ME 1:1 (volume ratio)	Cubic (*Pm*-3*m*)	4 °C/h	50 °C/48 h	2.64×10^4

Supplementary Note 1

Optimization of precursor solution concentration

We employed a solution with slightly lower than the up limit, primarily due to the following reason. For the control system, the heating rate is insufficient, approximately 2 °C h^{-1}, and we suggest using a faster heating rate (4 °C h^{-1}) to shorten the heating process. This requires us to balance the competition between nucleation and growth. The solution-based crystal growth model can be demonstrated using a dissolution-nucleation diagram[29]. As shown in Supplementary Fig. 5, the diagram contains two curves corresponding to the solubility curve and the supersaturation curve. It is noteworthy that the supersaturation curve is determined through the process of nucleation experiment. Specifically, the nucleation temperatures of different concentrations of perovskite solutions were recorded under certain heating conditions, and the upper limit for different concentrations was regarded as the supersaturation curve. The two curves divide the entire region into three parts: the stable zone, growth zone, and nucleation zone. Above the supersaturation curve, nucleation will occur spontaneously once thermodynamic and kinetic requirements are satisfied. The steady-state nucleation rate (j_0) on the crystal surface can be expressed as[30]:

$$j_0 = Aexp\left(\frac{-\Delta G^*}{kT}\right) \quad (1)$$

$$\Delta G^* = \Delta G_{Sol} - \Delta G_{Sur} \quad (2)$$

$$\Delta G_{Sol} = -RTln(S) \quad (3)$$

Where A represents the frequency factor of nucleation, ΔG^* is the free energy change and represents the activation energy for nucleation, k is the Boltzmann constant, T is the temperature, ΔG_{Sol} and ΔG_{Sur} are the Gibbs free energy change per unit bulk and surface, and S is saturation (the ratio of solute concentration to stable concentration).

As the solution concentration increases, the system approaches supersaturation, making ΔG_{Sol} and ΔG^* more negative. This leads to an exponential increase in the nucleation rate. When using the 2.5 mol L^{-1} solution, the system will exhibit lots of unwanted nucleus due to the increased nucleation density. Based on the dissolution-nucleation diagram, the nucleation temperature interval for the 2 mol L^{-1} solution ranges from 55 to 60 °C. To further reduces the duration of experiment, the initial temperature of the HFG solution can be set at 50 °C and then increased at a rate of 4 °C h^{-1}.

Supplementary Note 2

Crystal growth model.

We firstly consider the assumption of the growth model that diffusion is the key factor dominating the growth process of monocrystal. Crystal growth can be described by a two-step growth mode, which consists of the solute diffusion and the condensation of monomers on crystal surface. If solute diffusion limits the crystal growth rate, which is the favorable condition to obtain high-quality single crystals. On the other hand, if the monomers condensation rate is slower than the diffusion rate, the condensation rate becomes the limiting factor for crystal growth. Excess solute then leads to the formation of defect structures[30]. To quantitatively compare the criticality of solute diffusion and surface reaction, an effective factor η is defined as the ratio of the measured overall growth rate of the crystal to the growth rate when the crystal exposed to bulk solution. As the surface reaction becomes less important, $\eta \rightarrow 0$, the solute diffusion step dominates the whole growth process[31]. Obviously, the growth rate of thin monocrystals using the space-confined method (~0.001 g h^{-1}) is significantly smaller than that of bulk monocrystal in free solution (0.76 g h^{-1}), the latter yielding millimeter-size monocrystals within minutes[32]. Therefore, we believe that diffusion is the key factor dominating the growth of thin monocrystals (corresponding to an extremely small η), rather than surface reaction.

Crystal growth can be described by a two-step growth model[31,33], which consists of the solute diffusion (Eq. 1) and the condensation of monomers (Eq. 2) on crystal surface, as described by following equations:

$$J_D = 4D\pi r(C_b - C_i) \tag{4}$$

$$J_R = k(C_i - C_r) \tag{5}$$

where J_D is the flux of solute diffusion, J_R is the flux of surface reaction, D is diffusion coefficient, k is the rate of surface reaction, r is crystal size, C_b is bulk solution concentration, C_i is solute concentration at the crystal surface, C_r is solute solubility.

For simplicity, the thin monocrystal is modeled as two-dimentional circular crystal of which the lateral growth is dependent on the surface reaction and the Fick diffusion. The solute flux passing through the circular plane with radius x can be expressed by Fick's first law of diffusion:

$$J = -D\frac{\partial C}{\partial x} \quad (6)$$

where C is the solution concentration. Combining with the continuity equation, the concentration gradient changes with time that yields the Fick's second law of diffusion:

$$\frac{\partial C}{\partial t} = D\frac{\partial^2 C}{\partial x^2} \quad (7)$$

where t is the time.

To solve the partial differential equation, we take the following initial and boundary conditions for models with unlimited solute supply, i.e., models with infinite substrate size or the monomers at the edge of the substrate were provided infinitely:

$$t = 0, C = C_b$$

$$t \geq 0, x = +\infty, C = C_b \; or \; x = r_s, C = C_b$$

$$x = r, C = C_i$$

where r is the radius of crystal at time t, r_s is the radius of substrate, C_b is bulk solution concentration, C_i is solute concentration at the crystal surface. For models with specific size of substrate and limited solute supply, the following conditions are considered:

$$t = 0, C = C_b$$

$$t \geq 0, x = r, C = C_i$$

At the interface, the solute flux could be obtained by Eq. (2). On the basis of diffusion and surface reaction, C_i at specific t can be obtained by coupling Eq. (2) and (4) with a constant D, C_b, C_r, and k to denote the diffusion relationship between concentration and distance. Diffusion coefficient and bulk solution concentration are analyzed separately for diffusion calculation. The thin monocrystal is supposed to grow from a radius of 150 μm to 300 μm with k of 8×10^{-7} m s^{-1} and C_r of 0.7 mol L^{-1}. Since the diffusion is the limiting factor for crystal growth in this study, the change in radius with time can be given by:

$$\frac{dr}{dt} = \frac{Dv}{r}(C_b - C_r) \quad (8)$$

where v is the molar volume of the bulk crystal that could be calculated from M/ρ. The time for growth can be integrated from Eq. (5) with the specific D and C_b, and then being employed in growth model discussed above to gain the diffusion curve. For

diffusion calculation with different D, a constant C_b of 1.5 mol L^{-1} is set with varied D of 0.5, 1.0, 2.0, 3.0, and 4.0 $\times 10^{-10}$ m^2 s^{-1}. For the case with different C_b, a constant D of 2.0 $\times 10^{-10}$ m^2 s^{-1} is accepted with varied C_b of 1.0, 1.25, 1.5, 1.75, and 2 mol L^{-1}.

Supplementary Note 3

Solute flux.

We calculated the solute flux (J) of the thin monocrystal during steady-state growth, which is defined as the increment of mass per unit time (t) and area in contact with the solution. The thickness of a thin monocrystal remains constant due to substrate limitations, and the mass (m) can be easily calculated from its mass density (ρ), thickness (L), and surface area (A_s). The surface area of the thin monocrystal is accurately determined by the number of pixels from microscope images (A_f). The product of the total side length (a) and L of a thin monocrystal is the side area, which is also the area in contact with the solution. Thus, the solute flux can be calculated by the following formula:

$$J = \frac{dm}{A_f dt} = \frac{d(\rho L A_s)}{aLdt} = \frac{\rho d A_s}{adt} \tag{9}$$

Supplementary Note 4

Diffusion coefficient from model.

It is well known that the flux of solute diffusion is determined by the concentration gradient and the diffusion coefficient. Since the concentration gradient is known, to eliminate the influence of concentration on the diffusion coefficient, we use a precursor solution with a constant concentration (1.5 mol L^{-1}). To quantify the solute diffusion behavior during the growth of thin monocrystals, a model of solute/solvent diffusion was developed. The two precursor solutions are first diluted to 30 m mol L^{-1}, respectively. Due to the limitations of absorbance, the concentration of the completely diffused homogeneous solution is fixed at 0.1875 m mol L^{-1}. To initiate the diffusion process, 20 μL of the diluted solution is carefully injected into the bottom of a cuvette filled with 3.2 mL of the corresponding pure solvent. We use a UV–vis spectrometer to monitor the absorbance of diffusing solution at 10 mm from the bottom of the cuvette in real-time. To convert the absorbance measurements to concentration values, the Lambert-Beer law is applied, which states that the absorbance of a dilute solution is directly proportional to its concentration. Finally, we obtain the diffusion coefficients by fitting the concentration-time curves based on the analytical solution equation derived by Cagno, M. P. *et al.* (Eq. 7, see Ref. 34).

$$C(x,t) = \frac{A}{\sqrt{\pi}} \frac{e^{-x^2/2\sigma^2 + 4Dt}}{\sqrt{2\sigma^2 + 4Dt}} \tag{10}$$

where C is the concentration, A is the Fourier coefficients, x is the height of the monitoring position, σ is the width of the initial distribution, D is diffusion coefficient, t is time.

Supplementary Note 5

Diffusion-ordered spectroscopy (DOSY) is a well-established NMR technique used to probe the diffusion behavior of molecules in solution. Note the deuterated reagent is required to lock the magnetic field for NMR measurements, we attempted the commonly used deuterated reagents such as benzene, acetonitrile (ACN), chloroform, DMSO, etc into the system. We found that most of the deuterated solvents (benzene, chloroform, DMSO) cannot be compatible with the perovskite precursor solution, while only the addition of ACN at a volume ratio of 10% have an insignificant impact on the colloid nature. Previous reports have demonstrated that the ACN does not profoundly affect the coordination state in the 2-ME and GBL systems through UV−vis absorption spectra[35]. Furthermore, our DLS measurements show that the colloid size of 2-ME system is retained to be about 0.7 nm. We observed a slight reduction in the colloid size in GBL system (Supplementary Fig. 20), which we attribute to the dissociation of the lead-iodine complex[36]. Concerning the unconspicuous effect of ACN on colloidal chemistry, we operated DOSY measurements on these systems. Because of the introduction of deuterated ACN, the colloid size of the control system becomes smaller and therefore may give rise to over-estimate of diffusion coefficient by NMR measurements.

Supplementary Note 6

Diffusion coefficient from colloidal size.

We obtain the diffusion coefficients of solutes in different solvents by calculating Stokes-Einstein equation:

$$D = \frac{k_B T}{6\pi r \mu} \tag{11}$$

where k_B is the Boltzmann's constant, T is temperature, r is colloidal size from DLS measurement, and μ is viscosity of the solvent. In this work, the temperature of the HFG and control samples are 70 and 130 °C, and the viscosity of the HFG and control samples are 0.73 and 1.07 mPa s[37,38], respectively.

Supplementary References

[1] Alsalloum, A. Y. *et al.* Low-temperature crystallization enables 21.9% efficient single-crystal $MAPbI_3$ inverted perovskite solar cells. *ACS Energy Lett.* **5**, 657–662 (2020).

[2] Pan, D. *et al.* Deterministic fabrication of arbitrary vertical heterostructures of two-dimensional Ruddlesden-Popper halide perovskites. *Nat. Nanotechnol.* **16**, 159–165 (2021).

[3] Kahmann, S. *et al.* Negative thermal quenching in $FASnI_3$ perovskite single crystals and thin films. *ACS Energy Lett.* **5**, 2512–2519 (2020).

[4] Stoumpos, C. C. *et al.* Hybrid germanium iodide perovskite semiconductors: active lone pairs, structural distortions, direct and indirect energy gaps, and strong nonlinear optical properties. *J. Am. Chem. Soc.* **137**, 6804–6819 (2015).

[5] Liu, Y. *et al.* Multi-inch single-crystalline perovskite membrane for high-detectivity flexible photosensors. *Nat. Commun.* **9**, 5302 (2018).

[6] Shen, Y. *et al.* Centimeter-sized single crystal of two-dimensional halide perovskites incorporating straight-chain symmetric diammonium ion for X-ray detection. *Angew. Chem. Int. Ed.* **59**, 14896–14902 (2020).

[7] Singh, A. & Satapathi, S. Reversible thermochromism in all-inorganic lead-free $Cs_3Sb_2I_9$ perovskite single crystals. *Adv. Opt. Mater.* **9**, 2101062 (2021).

[8] Li, Y.-J. *et al.* Lead-free and stable antimony–silver-halide double perovskite $(CH_3NH_3)_2AgSbI_6$. *RSC Adv.* **7**, 35175–35180 (2017).

[9] Li, W.-G., Wang, X.-D., Liao, J.-F., Jiang, Y. & Kuang, D.-B. Enhanced on–off ratio photodetectors based on lead-free $Cs_3Bi_2I_9$ single crystal thin films. *Adv. Func. Mater.* **30**, 1909701 (2020).

[10] Liu, Y. *et al.* Large lead-free perovskite single crystal for high-performance coplanar X-ray imaging applications. *Adv. Opt. Mater.* **8**, 2000814 (2020).

[11] Li, W. *et al.* Zero-dimensional lead-free $FA_3Bi_2I_9$ single crystals for high-performance X-ray detection. *J. Phys. Chem. Lett.* **12**, 1778–1785 (2021).

[12] Jun, T. *et al.* Lead-free highly efficient blue-emitting $Cs_3Cu_2I_5$ with 0D electronic structure. *Adv. Mater.* **30**, 1804547 (2018).

[13] Keshavarz, M. *et al.* Tuning the structural and optoelectronic properties of $Cs_2AgBiBr_6$ double-perovskite single crystals through alkali-metal substitution. *Adv. Mater.* **32**, 2001878 (2020).

[14] Ge, C. *et al.* Centimeter-scale 2D perovskite $(PEA)_2PbBr_4$ single crystal plates grown by a seeded solution method for photodetectors. *RSC Adv.* **9**, 16779–16783 (2019).

[15] Ding, R. *et al.* A general wet transferring approach for diffusion-facilitated space-confined grown perovskite single-crystalline optoelectronic thin films. *Nano Lett.* **20**, 2747–2755 (2020).

[16] Rao, H.-S., Li, W.-G., Chen, B.-X., Kuang, D.-B. & Su, C.-Y. In situ growth of 120 cm^2 $CH_3NH_3PbBr_3$ perovskite crystal film on FTO glass for narrowband-photodetectors. *Adv. Mater.* **29**, 1602639 (2017).

[17] Chen, Y. X. *et al.* General space-confined on-substrate fabrication of thickness-adjustable hybrid perovskite single-crystalline thin films. *J. Am. Chem. Soc.* **138**, 16196–16199 (2016).

[18] Zhou, L. et al. Self-driven fast-response X-ray detectors based on vertical GaN p-n diodes. *IEEE Electr. Device L.* **40**, 1044–1047 (2019).

[19] Zhang, X., Zhu, T., Ji, C., Yao, Y. & Luo, J. In situ epitaxial growth of centimeter-sized lead-free $(BA)_2CsAgBiBr_7/Cs_2AgBiBr_6$ heterocrystals for self-driven X-ray detection. *J. Am. Chem. Soc.* **143**, 20802–20810 (2021).

[20] Gou, Z. et al. Self-driven X-ray photodetector based on ultrathin PbI_2 single crystal. *IEEE Electr. Device L.* **40**, 578–581 (2019).

[21] Wu, J. et al. Self-powered $FA_{0.55}MA_{0.45}PbI_3$ single-crystal perovskite X-ray detectors with high sensitivity. *Adv. Funct. Mater.* **32**, 2109149 (2021).

[22] Jiang, J. et al. Synergistic strain engineering of perovskite single crystals for highly stable and sensitive X-ray detectors with low-bias imaging and monitoring. *Nat. Photon.* **16**, 575–581 (2022).

[23] Ye, F. et al. High-quality cuboid $CH_3NH_3PbI_3$ single crystals for high performance X-ray and photon detectors. *Adv. Funct. Mater.* **29**, 1806984 (2018).

[24] Zhuang, R. et al. Highly sensitive X-ray detector made of layered perovskite-like $(NH_4)_3Bi_2I_9$ single crystal with anisotropic response. *Nat. Photon.* **13**, 602–608 (2019).

[25] Di, J. et al. Reveal the humidity effect on the phase pure $CsPbBr_3$ single crystals formation at room temperature and its application for ultrahigh sensitive X-ray detector. *Adv. Sci.* **9**, 2103482 (2022).

[26] Huang, Y. et al. A-site cation engineering for highly efficient $MAPbI_3$ single-crystal X-ray detector. *Angew. Chem. Int. Ed.* **58**, 17834–17842 (2019).

[27] Wei, W. *et al.* Monolithic integration of hybrid perovskite single crystals with heterogenous substrate for highly sensitive X-ray imaging. *Nat. Photon.* **11**, 315–321 (2017).

[28] Ji, C. *et al.* 2D Hybrid perovskite ferroelectric enables highly sensitive X-ray detection with low driving voltage. *Adv. Funct. Mater.* **30**, 1905529 (2019).

[29] Wang, W. *et al.* Electronic-grade high-quality perovskite single crystals by a steady self-supply solution growth for high-performance X-ray detectors. *Adv. Mater.* **32**, 2001540 (2020).

[30] Liu, Y. *et al.* Low-temperature-gradient crystallization for multi-inch high-quality perovskite single crystals for record performance photodetectors. *Mater. Today* **22**, 67–75 (2019).

[31] Karpiński, P. H. Importance of the two-step crystal growth model. *Chem. Eng. Sci.* **40**, 641–646 (1985).

[32] Saidaminov, M., *et al.* High-quality bulk hybrid perovskite single crystals within minutes by inverse temperature crystallization. *Nat. Commun*. **6**, 7586 (2015).

[33] Thanh, N. T., Maclean, N. & Mahiddine, S. Mechanisms of nucleation and growth of nanoparticles in solution. *Chem. Rev.* **114**, 7610–7630 (2014).

[34] Cagno, M. P. *et al.* Experimental determination of drug diffusion coefficients in unstirred aqueous environments by temporally resolved concentration measurements. *Mol. Pharmaceutics* **15**, 1488–1494 (2018).

[35] Deng, Y. *et al.* Tailoring solvent coordination for high-speed, room-temperature

blading of perovskite photovoltaic films. *Sci. Adv.* **5**, eaax7537 (2019).

[36] Chao, L. *et al.* Solvent engineering of the precursor solution toward large-area production of perovskite solar cells. *Adv. Mater.* **33**, 2005410 (2021).

[37] Shi, X., Li, C., Guo, H. & Shen, S. Density, viscosity, and excess properties of binary mixtures of 2-(methylamino)ethanol with 2-methoxyethanol, 2-ethoxyethanol, and 2-butoxyethanol from 293.15 to 353.15 K. *J. Chem. Eng. Data* **64**, 3960–3970 (2019).

[38] Chen, F. *et al.* Density, viscosity, speed of sound, excess property and bulk modulus of binary mixtures of γ-butyrolactone with acetonitrile, dimethyl carbonate, and tetrahydrofuran at temperatures (293.15 to 333.15) K. *J. Mol. Liq.* **209**, 683–692 (2015).
