# Supplementary material for: Universal growth of perovskite thin monocrystals from high solute flux for sensitive self-driven X-ray detection
Source: Nat Commun. 2024 Mar 16;15:2390. doi: 10.1038/s41467-024-46712-y (PMC10944467; doi:10.1038/s41467-024-46712-y)
Supplement: Supplementary file 3 — Description of Additional Supplementary Files [file 41467_2024_46712_MOESM3_ESM.pdf]

Description of Additional Supplementary Files

Supplementary Movie 1 The growth process of thin monocrystal of MAPbI3

Supplementary Movie 2 The growth process of thin monocrystal of PEA2PbBr4
